# Evidence of aerosol transmission of African swine fever virus between two piggeries under field conditions: a case study

**DOI:** 10.3389/fvets.2023.1201503

**Published:** 2023-06-01

**Authors:** Xiaowen Li, Zhiqiang Hu, Mingyu Fan, Xiaogang Tian, Weisheng Wu, Wenchao Gao, Lujie Bian, Xiaoxue Jiang

**Affiliations:** ^1^Xiajin New Hope Liuhe Agriculture and Animal Husbandry Co., Ltd., Dezhou, China; ^2^Shandong New Hope Liuhe Agriculture and Animal Husbandry Technology Co., Ltd. (NHLH Academy of Swine Research), Dezhou, China; ^3^Shandong Engineering Laboratory of Pig and Poultry Healthy Breeding and Disease Diagnosis Technology, Qingdao, China; ^4^China Agriculture Research System-Yangling Comprehensive Test Station, Xianyang, China

**Keywords:** ASFV, aerosol, air outlet, air inlet, dust

## Abstract

African swine fever (ASF) is a devastating and economically significant infectious disease that has caused enormous losses in the commercial pig sector in China since 2018. The primary transmission routes of the African swine fever virus (ASFV), the causative agent of ASF, are direct pig-to-pig contact or indirect contact with virus-contaminated objects. While aerosol transmission of ASFV has been previously reported under experimental conditions, no reports have described it under field conditions. In this case study, aerosol-associated samples were collected over a monitoring period of 24 days in an ASFV-positive farm. A complete and clear chain of ASFV transmission through aerosols was observed: pigs in Room A on Day 0-aerosol in Room A on Day 6-dust of air outlets in Room A on Day 9-outdoor aerosols on Day 9-dust of air inlets in Room B on Day 15-aerosols/pigs in Room B on Day 21. Furthermore, a fluorescent powder experiment confirmed the transmission of dust from Room A to Room B. This study represents the first report providing evidence of aerosol transmission of ASFV under field conditions. Further research is needed to study the laws of aerosol transmission in ASFV and develop effective strategies such as air filtration or disinfection to create a low-risk environment with fresh air for pig herds.

## Introduction

ASF is an acute, febrile, highly contagious infectious disease listed by the World Organization for Animal Health (WOAH) as a notifiable disease (1), with a morbidity and mortality rate as high as 100% in domestic pigs when it first occurred in China (2, 3). ASFV, the causative agent of ASF, belongs to the *Asfivirus* genus within the *Asfarviridae* family. It was first reported in 1921 in East Africa, and rapidly spread to other African countries (4). ASFV outbreak was first reported in China in 2018 (5, 6), and it caused the death of 1.193 million pigs by November 2021 (7).

The major transmission routes of ASFV include direct pig-to-pig contact or indirect contact with virus-contaminated objects, such as excretory materials (8, 9), feed (10), water (10, 11), and needles (2). To prevent ASFV diffusion and maintain the health of pig populations, a partitioned approach has been developed and proven effective. This approach involves improving the biosecurity level of pig farms to reduce the risk of ASF introduction, strengthening monitoring procedures for early detection, culling and removing positive groups to eliminate the risk, and implementing strict disinfection measures to eliminate pollution sources and interrupt transmission routes (12). However, with the emergence of mutant ASFV strains, further improvements in this strategy are necessary.

Aerosol transmission is another important route for ASFV spread. Aerosol transmission occurs when susceptible animals inhale pathogen-carrying particles with a diameter of less than 5 μm (13). Aerosols typically contain suspended solid or liquid particles in the air (13). While a study in 1977 showed ASFV transmission up to a distance of 2.3 meters in a confined space, no detection of ASFV in the air was reported (14). However, since 2012, air sampling methods have proven effective in detecting ASFV particles in the air. Although only a few experimental studies (15, 16) have reported aerosol transmission of ASFV, no field studies have been conducted to date. In this study, we present evidence that aerosols carrying ASFV can be found in piggeries under field conditions.

## Method

### Farm description

The farm in this study is a commercial farm located in Shandong Province. It is equipped with automatic feeding systems, automatic drinking water systems, and comprehensive biosecurity measures. External biosecurity standards require that all individuals and materials entering the farm must undergo bathing or disinfection procedures and test negative for ASFV before entry. Internal biosecurity standards involve dividing the farm area into one living area and four breeding areas, each with a one-way gate at the entrance. People entering the breeding areas must take a bath and change into disinfected clothes, and all materials entering these areas must undergo high-temperature treatment or be soaked in disinfectant. Furthermore, farmers are dedicated to specific herds and do not cross between them. Therefore, there was no any intersection of feed, water, materials or farmers between rooms.

**Figure 1:**
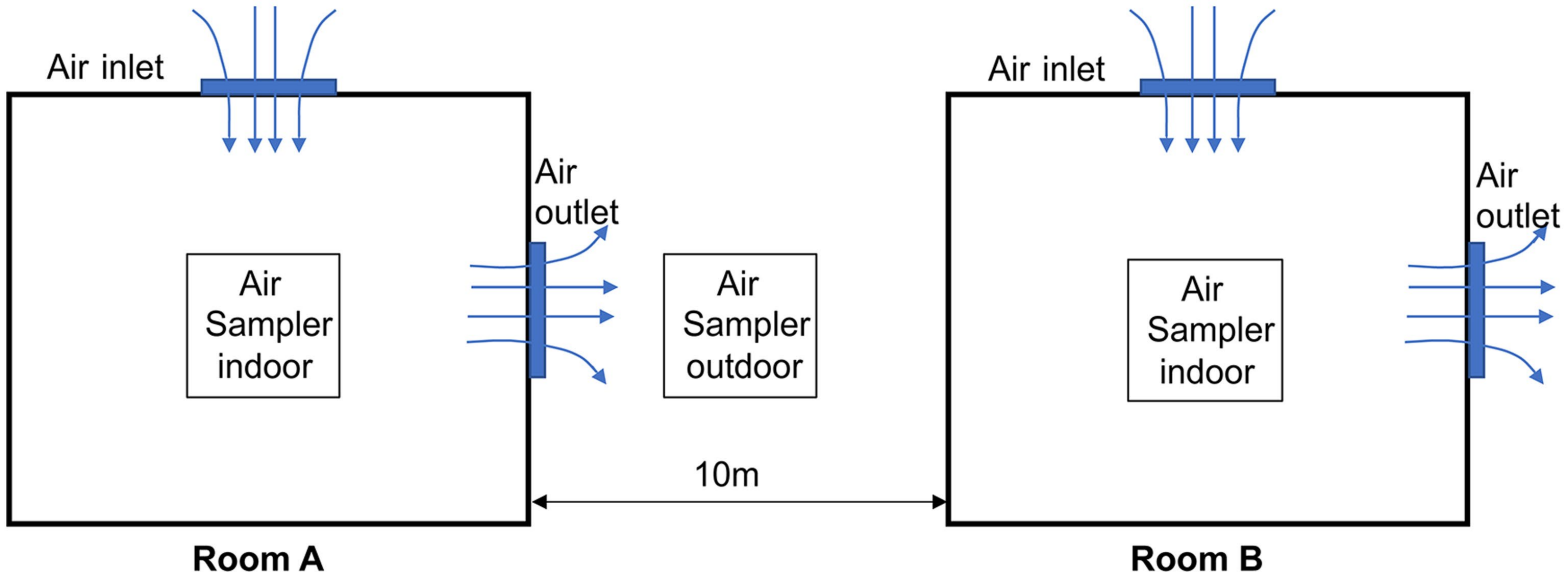
Schematic map of two ASFV-positive piggeries and areas of aerosol-associated samples.

ASFV was detected on this farm in December 2021. The farm consists of two delivery rooms, Room A and Room B, each housing 60 sows. These rooms are adjacent to each other, with a distance of 10 meters between them, as shown in Figure 1. The ventilation mode during winter in Room A and B is longitudinal, as commonly used in northern Chinese pig farms during the winter season. It is worth noting that this ventilation mode is smaller than that used in the summer. The first ASFV-positive sow was detected in Room A and this day were defined as Day 0. Subsequently, whole-piggery samples were collected every 6 days, and ASFV-positive sows were removed from the herd, while the remaining sows were continuously tested. All sows in both Rooms A and B were sampled using serum and tested by qPCR, with a Cq value of <40 considered positive. Additionally, a whole-piggery-sampling was performed in Room B on Day 21.

### Collection of different samples

Indoor aerosol samples were collected using the MD8 air scan sampling device (Sartorius, Nieuwegein, Netherlands) at an air speed of 50 m^3^/min for 20 min. Sterile gelatine filters of a pore size of 3 mm and a diameter of 80 mm (type 17,528-80-ACD, Sartorius) were then dissolved in 5 mL of normal saline. Outdoor aerosol samples were collected using the GR1356-Microbial concentration sampler (Qingdao Guorui Liheng Environmental Protection Technology Co. LTD, Qingdao, China) at an air speed of 120 m^3^/min for 6 h. All microorganisms were subsequently gathered in 5 mL of normal saline. As depicted in Figure 1, the sampler was positioned in the middle of the two rooms.

Dust samples from the surface of air outlets and air inlets were collected by wiping them with a gauze (10 cm × 10 cm), and then eluting them with 10 mL of normal saline. All samples were collected once every 3 days and tested by qPCR, with a Cq value of <40 considered positive.

### qPCR

All the samples were tested using qPCR following the previously described method (17). Briefly, 300 μL of serum, aerosol solution, or dust solution were subjected to DNA extraction using the Automatic nucleic acid extractors (NPA-96E) from Bioer Technology Co., Ltd. (Hangzhou, China). Subsequently, 5 μL of the extracted DNA was utilized for qPCR detection, which was performed on a Step One Plus instrument (ABI) using the PerfectStart^®^ II Probe qPCR SuperMix (TransGen Biotech, China) according to the manufacturer’s instructions. Specific primers for the ASFV B646L gene were designed based on the ASFV isolate Pig/HLJ/18 (GenBank: MK333180.1) (5) and used for qPCR: 5’-AAAATGATACGCAGCGAAC-3′(forward), 5’-TTGTTTACCAGCTGTTTGGAT-3′ (reverse), and 5’-FAM-TTCACAGCATTTTCCCGAGAACT-BHQ1-3′ (probe) (17). The detection limit of the qPCR assay was determined to be 2.5 copies/μL of the ASFV genome. The results of qPCR were recorded as quantification cycle values (Cq values), and a Cq value of <40 was considered as a positive result.

### Fluorescent powder experiment

Fluorescent powder, a dust-like substance, is commonly employed to simulate the movement and dispersion of dust or aerosols. It has been utilized in various settings, including the assessment of contamination and the effectiveness of cleaning procedures in theatres during the COVID-19 pandemic (18). In this study, fluorescent powders were placed near the four outlets in Room A. After a 3 day period, dust samples were collected from the surfaces of the air outlets in Room A and the air inlets in Room B using the previously described method. Subsequently, gauzes were spread out and photographed under dark conditions to visualize the presence and distribution of the fluorescent powder.

## Results

In this field study, we monitored the detection of ASFV in aerosol-associated samples in an ASFV-positive farm over a 24 day period following the confirmation of the first case of ASFV-positive pigs.

**Figure 2:**
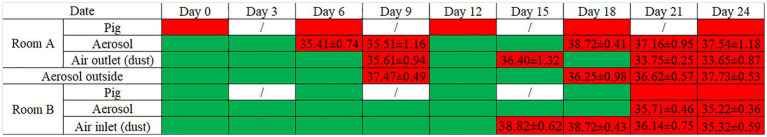
ASFV detection in pigs and aerosol-associated samples in Rooms A and B. Cells in red color: ASFV-positive; cells in green color: ASFV-negative; number in red cells: Cq value of qPCR (Mean ± SD); “/”: no detection.

As shown in Figure 2, pigs of whole herds in Rooms A and B have been detected throughout the monitoring period, from Day 0 to Day 24. The Cq values of positive pigs were shown in Supplementary Table S1. In Room A, aerosol samples initially tested positive on Day 6, and continued to be positive until Day 24, despite negative samples on Days 12 and 15. Interestingly, Cq values of aerosol samples on Day 6 and 9 were lower compared to those on Day 18, 21, and 24, possibly indicating the removal of most ASFV-positive pigs in the later stage. Dust samples collected from air outlets were the last to be test positive on Day 9 among all sample types, and remained positive until Day 24. Furthermore, from Day 15 on, a downward trend in Cq values was observed from Day 15 onwards, suggesting the accumulation of the virus in the dust. These findings suggest that during an ASFV outbreak, ASFV particles excreted from infected pigs can be present in suspended aerosols and settling dust.

In Room B, as shown in Figure 2, ASFV-positive dust collected from air inlets was first detected on Day 15. Notably, there was a significant drop in the Cq value on Day 21. On the same day, pigs and aerosol samples were also detected as positive for ASFV, suggesting a possible association with the presence of positive dust in the air inlets.

Figure 2 also reveals that outdoor aerosol samples first tested positive on Day 9, coinciding with the collection of dust samples from air outlets in Room A. Additionally, outdoor aerosol samples remained positive until Day 24.

**Figure 3:**
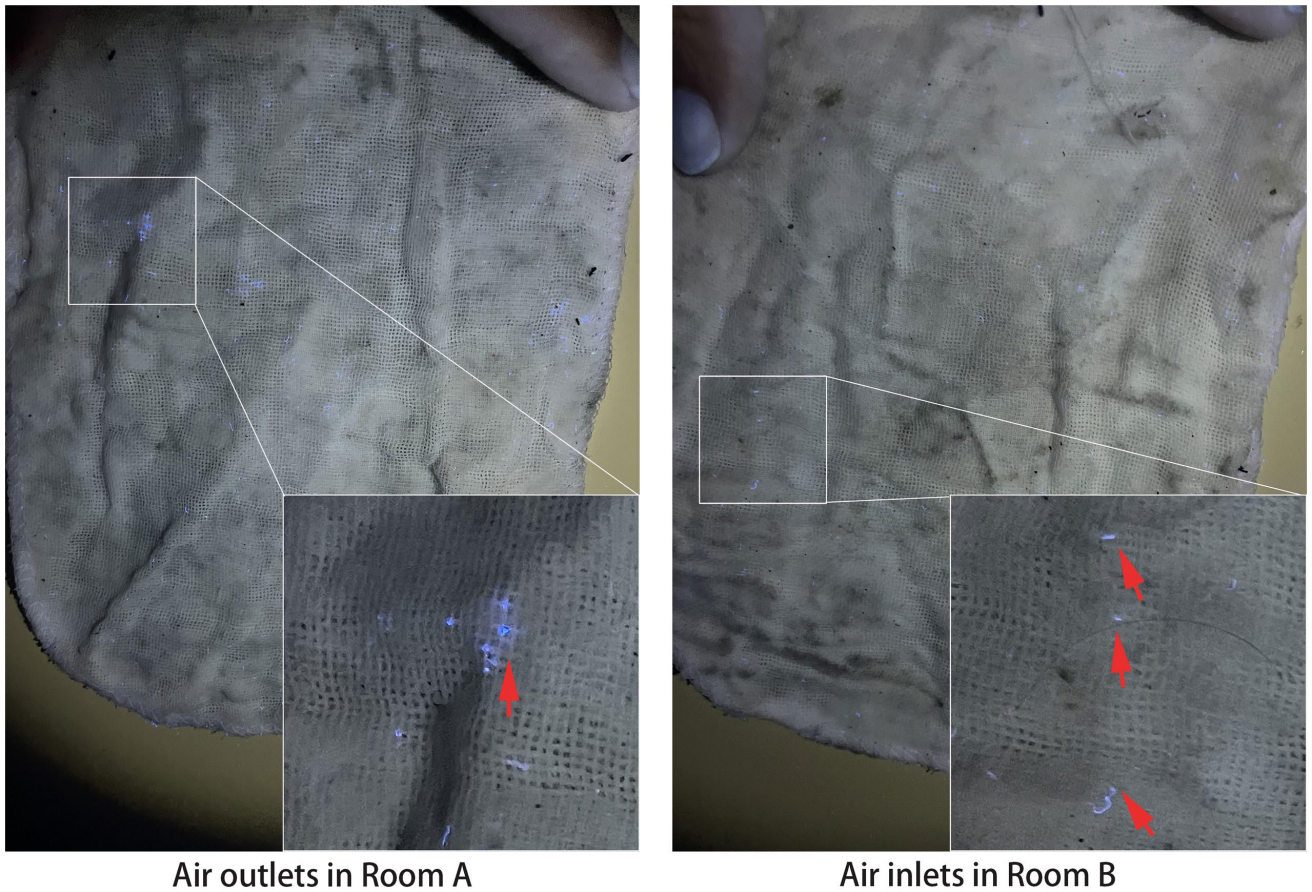
Fluorescent spots of gauzes from Rooms A and B. Upward arrows point to the blue fluorescent spots.

To investigate whether the dust in the air inlets of Room B originated from Room A, fluorescent powder was used to trace the dust trajectory from Room A. As depicted in Figure 3, 3 days later, fluorescent spots were observed on gauzes from both the air outlets in Room A and the air inlets in Room B, indicating the potential transmission of dust from Room A to Room B.

## Discussion and conclusion

Aerosol transmission of infectious agents is widely recognized as one of the most challenging routes to prevent and control (19), particularly in commercial farms. Several swine virus, including foot-and-mouth disease virus (FMDV) (20), porcine reproductive and respiratory syndrome virus (PRRSV) (21), porcine epidemic diarrhea virus (PEDV) (22), and influenza A virus (IAV) (23), have been reported to spread through aerosols. However, the aerosol transmission of ASFV has been has been a subject of debate, with limited evidence from experimental studies (15, 16). Moreover, the current prevention strategies do not specifically address aerosol transmission risks. In this case study, we have found evidence of aerosol transmission of ASFV between two piggeries under field conditions.

By analyzing the dates of the first detection of different samples, we have identified a complete and clear chain of ASFV transmission via aerosols: infected pigs in Room A release aerosols, which contaminate the dust on air outlets in Room A. Subsequently, outdoor aerosols become contaminated, leading to the deposition of contaminated dust on air inlets in Room B, resulting in the transmission of aerosols and/or infected pigs to Room B. This represents a novel transmission route of ASFV between piggeries. The source of ASFV-positive aerosols is likely the excretions and secretions of ASFV-positive pigs, including urine, sneezes and feces (7). Previous research has proven that the positive aerosols were associated with viruses in feces (15), supporting our hypothesis. Dust also plays a crucial role in spreading ASFV particles among piggeries, although it is often overlooked by farmers due to its presence in hard-to-reach locations.

The presence of ASFV-positive dust poses a significant risk to the entire herd, especially if it becomes agitated due to factors such as sudden changes in wind direction or feeding activities in piggeries. In addition, contaminated feed, which has been identified as a primary risk for ASFV transmission (24, 25), can contribute to the presence of ASFV in dust. Therefore, timely removal of dust in piggeries is crucial for the control and prevention of ASFV transmission.

Outdoor aerosol detection is another critical factor to consider. Due to the wide range of outdoor aerosols, detecting their presence can be challenging. In our study, we employed the GR1356-Microbial concentration sampler, which allowed continuous aerosol collection for 6 h at a time. Outdoor aerosol samples remained positive until Day 24 during the experimental period, highlighting the persistent risk of aerosol transmission. Air filtration systems have been proven effective in preventing aerosol transmission of other pathogens, such as PRRSV (26). Therefore, integrating air filtration systems into the biosecurity measures against ASFV and other pathogens is recommended, especially for small farms with poor biosecurity practices in China (7).

The travel distance of viral aerosols is a significant concern. Wilkinson et al. demonstrated that ASFV can be transmitted through the air with a maximum distance of 2.3 meters (14). In our study, the distance between Rooms A and B was 10 meters, indicating that ASFV aerosols traveled at least 10 m. The difference in transmission distance could be attributed to environmental factors such as the outdoor temperature and the wind speed, as the temperature was below 4°C and strong winds were prevalent in northern China in winter. Furthermore, the transmission distance might also be influenced by the strain of the virus. In this case study, the ASFV strain belonged to Genotype I, causing mild onset of infection and chronic disease (27), and previous research has suggested that lower virulence strains tend to be highly transmissible (28). Further research is needed to investigate the transmission distance of ASFV aerosols.

In conclusion, this case study provides evidence of aerosol transmission of ASFV under field conditions, expanding our understanding of ASFV transmission routes. We emphasize the importance of considering air inlet and outlet filtration, strengthening air disinfection measures, and reducing dust levels in pig farms to create a low-risk environment with fresh air for pig herds.

## Data availability statement

The original contributions presented in the study are included in the article/Supplementary material, further inquiries can be directed to the corresponding author.

## Ethics statement

Ethical review and approval were not required for the animal study because the manuscript is a case study of spontaneous disease. Written informed consent was obtained from the owners for the participation of their animals in this study.

## Author contributions

XL conceived and designed the analyzation method. XL, ZH, and MF collected and analyzed the data, and also wrote the original draft and reviewed and edited the manuscript. XT, WW, WG, LB, and XJ contributed to collect samples from field farms and the data of qPCR test. All authors contributed to the article and approved the submitted version.

## Funding

This work was supported by Taishan Industry Leadership Talent Project of Shandong province in China, National Key R&D Program of China (2021ZD0113800) and the earmarked fund for CARS (CARS-35).

## Conflict of interest

XL, ZH, MF, and WW were employed by Xiajin New Hope Liuhe Agriculture and Animal Husbandry Co., Ltd. XL, ZH, MF, XT, WG, LB, and XJ were employed by Shandong New Hope Liuhe Agriculture and Animal Husbandry Technology Co., Ltd. (NHLH Academy of Swine Research).

## Publisher’s note

All claims expressed in this article are solely those of the authors and do not necessarily represent those of their affiliated organizations, or those of the publisher, the editors and the reviewers. Any product that may be evaluated in this article, or claim that may be made by its manufacturer, is not guaranteed or endorsed by the publisher.
