# Supplementary material for: Evidence of aerosol transmission of African swine fever virus between two piggeries under field conditions: a case study
Source: Front Vet Sci. 2023 Jun 1;10:1201503. doi: 10.3389/fvets.2023.1201503 (PMC10267313; doi:10.3389/fvets.2023.1201503)
Supplement: Supplementary file 1 [file Table_1.docx]

**Table S1** Cq values of African swine fever virus detection of pigs in Room A and Room B

| Date | Day 0 | Day 3 | Day 6 | Day 9 | Day 12 | Day 15 | Day 18 | Day 21 | Day 24 |
| --- | --- | --- | --- | --- | --- | --- | --- | --- | --- |
| Room A | 29.92/ 35.12±0.93/ 36.25±0.88 (n=3) | / | 36.53±0.38 (n=1) | / | 36.21±0.28/ 34.15±0.95 (n=2) | / | 37.31±0.42 (n=1) | / | 32.12±0.87 (n=1) |
| Room B | No | / | No | / | No | / | No | 37.49±0.47/ 36.53±0.37 (n=2) | 35.55±0.72/ 33.67±0.79 (n=2) |

“n” means the number of positive pigs; “No” means all pigs negative; “/” means no detection on that day.
